# An Age-Specific Atlas for Delineation of White Matter Pathways in Children Aged 6-8 Years

**DOI:** 10.1089/brain.2021.0058

**Published:** 2021-08-23

**Authors:** Arthur P.C. Spencer, Hollie Byrne, Richard Lee-Kelland, Sally Jary, Marianne Thoresen, Frances M. Cowan, Ela Chakkarapani, Jonathan C.W. Brooks

**Affiliations:** a Clinical Research and Imaging Centre, University of Bristol, Bristol, UK; b Translational Health Sciences, Bristol Medical School, University of Bristol, Bristol, UK; c Faculty of Medicine, Institute of Basic Medical Sciences, University of Oslo, Oslo, Norway; d Department of Paediatrics, Imperial College London, London, UK; e School of Psychology, University of East Anglia, Norwich, UK

**Keywords:** White matter, atlas, diffusion MRI, tractography

## Abstract

**Introduction:**

Diffusion MRI allows non-invasive assessment of white matter connectivity in typical development and of changes due to brain injury or pathology. Probabilistic white matter atlases allow diffusion metrics to be measured in specific white matter pathways, and are a critical component in spatial normalisation for group analysis. However, given the known developmental changes in white matter it may be sub-optimal to use an adult template when assessing data acquired from children.

**Methods:**

By averaging subject-specific fibre bundles from 28 children aged from 6 to 8 years, we created an age-specific probabilistic white matter atlas for 12 major white matter tracts. Using both the newly developed and Johns Hopkins adult atlases, we compared the atlas to subject-specific fibre bundles in two independent validation cohorts, assessing accuracy in terms of volumetric overlap and measured diffusion metrics.

**Results:**

Our age-specific atlas gave better overall performance than the adult atlas, achieving higher volumetric overlap with subject-specific fibre tracking and higher correlation of FA measurements with those measured from subject-specific fibre bundles. Specifically, estimates of FA values for cortico-spinal tract, uncinate fasciculus, forceps minor, cingulate gyrus part of the cingulum and anterior thalamic radiation were all significantly more accurate when estimated with an age-specific atlas.

**Discussion:**

The age-specific atlas allows delineation of white matter tracts in children aged 6-8 years, without the need for tractography, more accurately than when normalising to an adult atlas. To our knowledge, this is the first publicly available probabilistic atlas of white matter tracts for this age group.

## 1 Introduction

Tract-level analysis of diffusion weighted imaging (DWI) data is used extensively to investigate white matter in both typical (Asato et al., 2010; Lebel et al., 2008; Hüppi and Dubois, 2006) and atypical brain development (for a review, see (Dennis and Thompson, 2013)). In children and adolescents, atypical brain development may lead to physical and intellectual disabilities including e.g. cerebral palsy (CP) (Arrigoni et al., 2016), autistic spectrum behaviours (Dimond et al., 2019; Ameis and Catani, 2015) and attention deficit hyperactivity disorder (Konrad and Eickhoff, 2010). Diffusion metrics such as fractional anisotropy (FA), mean diffusivity, radial diffusivity and axial diffusivity (Assaf and Pasternak, 2008) are sensitive to changes in the underlying white matter structure. These metrics are widely investigated in studies of brain development (Dennis and Thompson, 2013; Lebel et al., 2008), as well as having clinical relevance in patient cohorts (Assaf et al., 2019; Assaf and Pasternak, 2008; Horsfield and Jones, 2002).

To measure tract-level diffusion metrics, white matter tracts can be delineated by registering to a standard template with a probabilistic atlas of tract locations. Using a white matter atlas eliminates the need for computationally intensive methods of delineating tracts by segmenting streamlines generated by tractography (Sydnor et al., 2018; Zhang et al., 2018; Wassermann et al., 2010; Lawes et al., 2008; Wakana et al., 2007). This is beneficial in clinical settings or when studying large datasets. Additionally, data which have been acquired with shorter, more simplistic diffusion tensor acquisitions may not facilitate accurate tractography. Such acquisitions may be favoured in an effort to minimise scan times (and therefore minimise risk of movement during the scan) when studying children, including those with disabilities who would benefit from investigating white matter diffusion properties (Phan et al., 2018).

The widely used Johns Hopkins University (JHU) white matter tract atlas (Hua et al., 2008) is constructed from adult data. Numerous developmental studies demonstrate white matter alterations continuing into adolescence (Simmonds et al., 2014; Hagmann et al., 2010; Lebel et al., 2008; Cascio et al., 2007), and white matter development varies widely across the brain (Lebel et al., 2019), therefore an atlas constructed from adult scans is by design and definition not representative of children. There are several publicly available age-specific structural templates (Richards et al., 2016; Sanchez et al., 2012; Fonov et al., 2011; Altaye et al., 2008), however none of these provide diffusion data.

In this study, we used robust tract reconstruction protocols (Hua et al., 2008; Wakana et al., 2007) to develop an age-specific probabilistic white matter atlas for 12 major tracts in children aged 6-8 years. To assess whether this atlas accurately delineates tracts, we measured both volumetric overlap and FA values sampled by the tract mask in comparison with subject-specific tractography-based tract delineation. We then assessed the utility of this age-specific tract atlas by comparing it to results obtained using an adult atlas (JHU). The atlas was then further validated against an open data source (i.e. different scanner and acquisition protocol), and against a different tractography algorithm.

As a demonstration, we then investigated tract-level differences in children treated with therapeutic hypothermia (TH) for neonatal encephalopathy (NE) at birth, compared with healthy controls, and compared results obtained using the age-specific atlas to those from the JHU atlas. The children who had TH, do not have CP and are in mainstream education still exhibit significantly reduced performance on cognitive tests (Lee-Kelland et al., 2020; Jary et al., 2019) and have slower reaction times and reduced visuo-spatial processing abilities (Tonks et al., 2019) compared to the typically developing controls.

## 2 Material and Methods

### 2.1 Participants

Ethics approval was obtained from the North Bristol Research Ethics Committee and the Health Research Authority (REC ID: 15/SW/0148). Informed and written consent was obtained from the parents of participants before collecting data. The cohort was made up of 36 healthy children aged 6-8 years with no evidence of neurological disease, originally recruited as controls for a study of the long-term effects of TH (“CoolMRI”) on behavioural and imaging outcomes. The 36 controls were split randomly into 28 atlas and 8 validation subjects such that the group were matched for age, sex, socio-economic status (SES) and full-scale intelligence quotient (FSIQ). A further 15 validation subjects were obtained from an open data source (see Section 2.5.4). For the demonstrative case study, data from 33 children treated with TH following NE at birth were compared to the 36 control children.

### 2.2 Image Acquisition

DWI data were acquired with a Siemens 3 tesla Magnetom Skyra MRI scanner at the Clinical Research and Imaging Centre (CRiCBristol), Bristol, UK. Subjects were placed supine within the 32-channel receive only head-coil by an experienced radiographer, and head movement minimised by means of memory-foam padding. Children wore earplugs and were able to watch a film. A multiband echo-planar imaging sequence was used with the following parameters: TE = 70 ms; TR = 3150 ms; field of view 192 × 192 mm; 60 slices; 2.0 mm isotropic voxels; phase encoding in the anterior-posterior direction, in-plane acceleration factor = 2 (Griswold et al., 2002), through-plane multi-band factor = 2 (Setsompop et al., 2012*a*, *b*; Moeller et al., 2010). For the purpose of data averaging and eddy-current distortion correction, two sets of diffusion weighted images were acquired with b = 1,000 s mm^-2^ in 60 diffusion directions, equally distributed according to an electrostatic repulsion model, as well as 8 interspersed b = 0 images, with one data set acquired with positive phase encoding steps, then repeated with negative steps (so-called, “blip-up”, “blip-down”), giving a total of 136 images.

### 2.3 Quality Control

The quality of the diffusion data was assessed using the EddyQC tool (Bastiani et al., 2019) from FSL (Smith et al., 2004). This provides several measures of the amount of movement and eddy current induced distortion present in the data. For each participant, metrics were normalised, then the root-mean-square was calculated, giving a score which increases monotonically with the amount of movement and eddy current distortion. Scans were rejected if their score was more than one standard deviation above the mean of all participants.

### 2.4 Image Processing & Atlas Construction

DWI data were corrected for eddy current induced distortions and subject movements using EDDY (Andersson and Sotiropoulos, 2016) and TOPUP (Andersson et al., 2003), part of FSL. Subsequent DWI processing and tractography steps were performed using MRtrix (Tournier et al., 2019). The response function (the DWI signal for a typical fibre population) was estimated from the data (Tournier et al., 2013). The fibre orientation distribution (FOD) was then calculated by performing constrained spherical deconvolution of the response function from the measured DWI signal (Tournier et al., 2007). Deterministic tractography was run in each subject using the “SD Stream” algorithm (Tournier et al., 2012). Streamlines were seeded randomly in the brain and generated with a step size of 0.2 mm, then terminated if the FOD amplitude dropped below 0.2 or the angle between successive steps exceeded 40 degrees. 10 million streamlines were generated, which were then filtered to 1 million using spherical-deconvolution informed filtering of tractograms (Smith et al., 2013) to give better reconstruction of FODs, improving anatomical accuracy.

**Figure 1:**
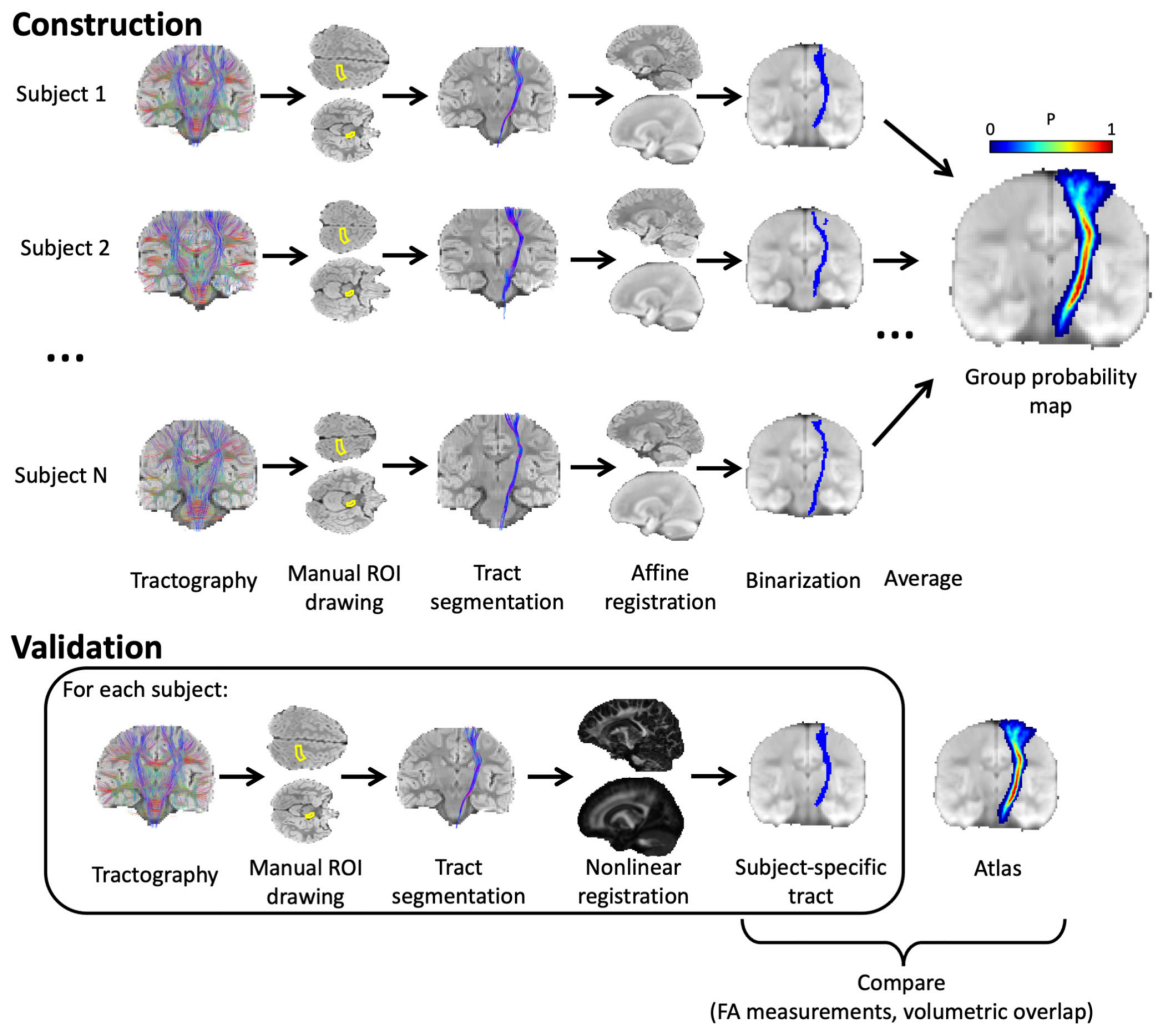
Methodology for generating probabilistic tract maps from whole-brain tractography data, shown here for the corticospinal tract (CST). ROIs were manually drawn in each subject, as defined by (Wakana et al., 2007) (in the case of the CST, inclusion ROIs were drawn in the cerebral peduncle and the primary motor cortex), and tracts were segmented by including streamlines passing through the inclusion ROIs. Streamlines were transformed to standard space (JHU template) and a binary mask was created for each subject indicating all voxels containing streamlines. The average of these masks (across N = 28 subjects) gives the probability map.

**Figure 2:**
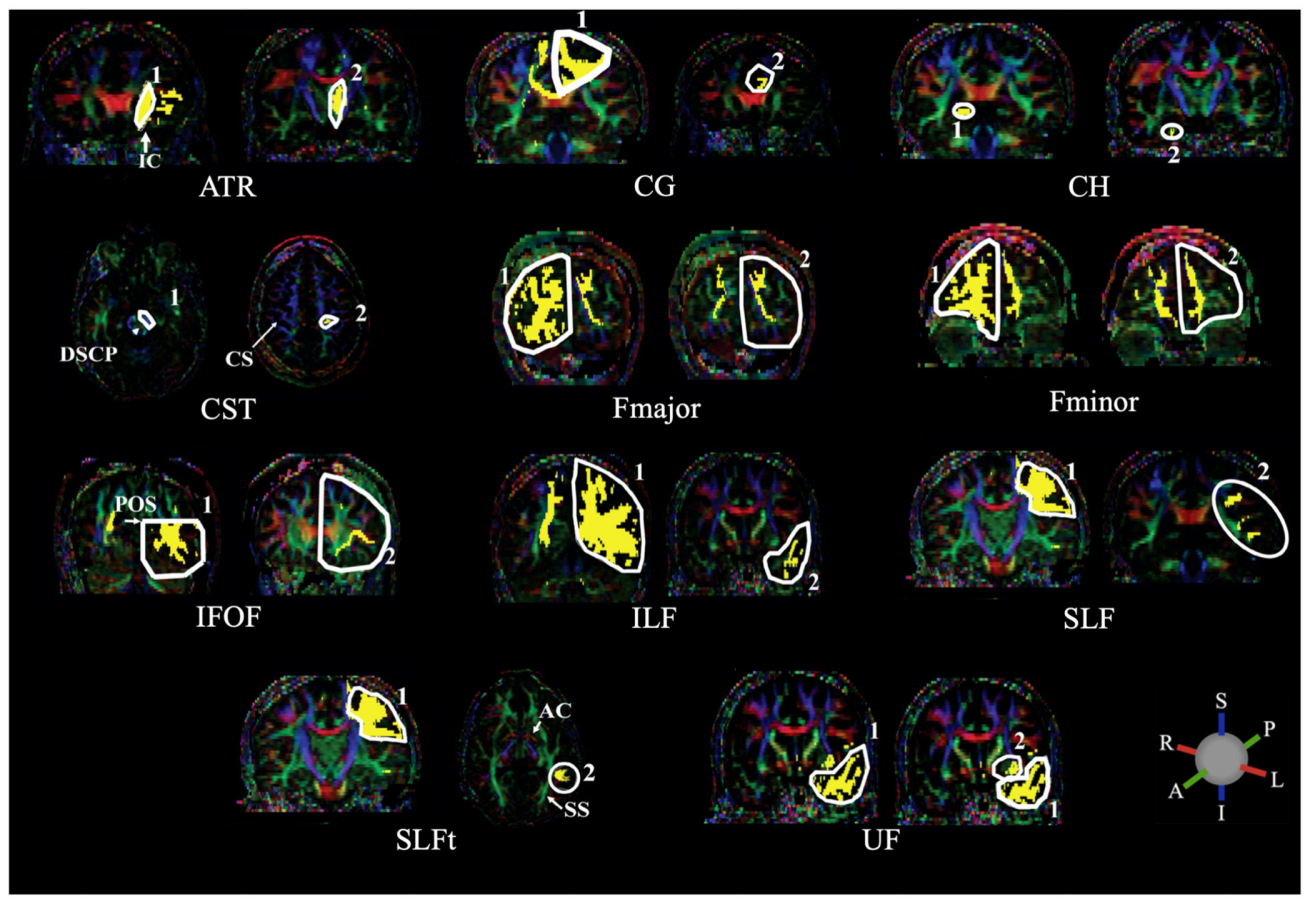
ROIs used to delineate the following major white matter tracts: anterior thalamic radiation (ATR); cingulate gyrus part of the cingulum (CG); hippocampal part of the cingulum (CH); cortico-spinal tract (CST); forceps major (Fmajor); forceps minor (Fminor); inferior fronto-occipital fasciculus (IFOF); inferior longitudinal fasciculus (ILF); superior longitudinal fasciculus (SLF); temporal part of the superior longitudinal fasciculus (SLFt); uncinate fasciculus (UF). Streamlines are included in a given tract if they pass through both 1 AND 2. The following abbreviations indicate anatomical landmarks used to draw the ROIs: internal capsule (IC); decussation of the superior cerebellar peduncle (DSCP); central sulcus (CS); parieto-occipital sulcus (POS); anterior commissure (AC); sagittal stratum (SS). ROIs are drawn in white with streamlines in yellow, overlaid on FA images with principal diffusion directions indicated by the colour ball; blue = superior-inferior (S-I), green = anterior-posterior (A-P) and red = right-left (L-R). Adapted from Hua et al., 2008, with permission from Elsevier.

The process of generating probability maps from the whole-brain tractograms is summarised in Figure 1. White matter tracts were segmented from whole-brain tractograms using the protocols described in Wakana et al., whereby regions of interest (ROI) are drawn to include or exclude streamlines passing through them (Wakana et al., 2007). For a given tract, any streamlines which pass through all inclusion ROIs and no exclusion ROIs belong to that tract, and all other streamlines are removed. Inclusion and exclusion ROIs were manually drawn in each subject to delineate 12 major fibre tracts: anterior thalamic radiation (ATR); cingulate gyrus part of the cingulum (CG); hippocampal part of the cingulum (CH); cortico-spinal tract (CST); forceps major (Fmajor); forceps minor (Fminor); inferior fronto-occipital fasciculus (IFOF); inferior longitudinal fasciculus (ILF); superior longitudinal fasciculus (SLF); temporal projections of the superior longitudinal fasciculus (SLFt); uncinate fasciculus (UF); and the fornix. The locations of ROIs for all tracts apart from the fornix are described in Wakana et al. as shown in Figure 2 (Hua et al., 2008; Wakana et al., 2007).

**Figure 3:**
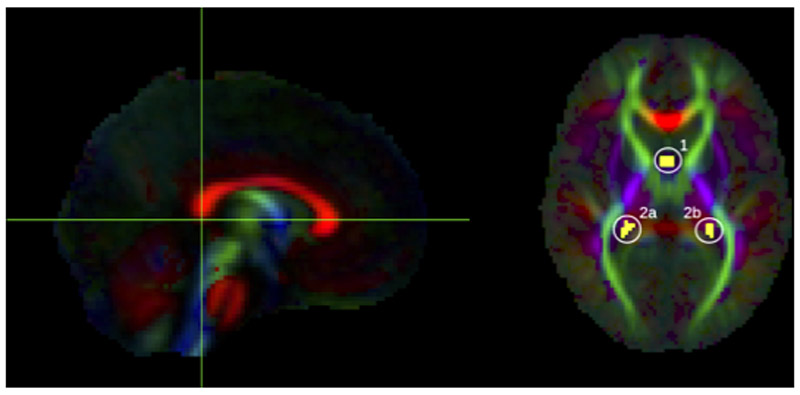
ROIs used to delineate the fornix, shown here on the group FA template. Yellow voxels contain streamlines which pass through the body of the fornix (1) AND bilateral posterior limbs of fornix (2a OR 2b).

To delineate the fornix, streamlines were included which pass through the body of the fornix and either of the posterior limbs which project to the hippocampus (Figure 3). These were isolated by first selecting an axial level at the lower edge of the splenium of the corpus callosum, as seen in the mid-sagittal plane (Figure 3, left); in this axial level, the first ROI was drawn around the body of the fornix. Viewing the streamlines which are delineated by the first ROI, additional bilateral ROIs were defined to include only those which project posteriorly from the fornix body (Figure 3, right).

For spatial normalisation, the average diffusion weighted image (aDWI), created for each subject by averaging all DWI images, was registered to the JHU aDWI template by 12-degree of freedom affine registration using FSL’s FLIRT (Jenkinson et al., 2002) (note that affine registration was used here when generating the atlas, in order to maintain inter-subject variability in tract anatomy, whereas nonlinear registration was used in later validation steps in order to prevent bias towards the age-specific template). The resulting transformation was then applied to the segmented streamlines. Any voxel containing one or more of these streamlines was then labelled, to create a binary mask for the tract for each individual. The average, across 28 subjects, of these binary masks was taken to give a probability map for each tract. The aDWI was then created for the group by averaging transformed aDWIs from all 28 subjects. To create the group FA image, the affine transformation for each subject, given by registration of the aDWI images (described above), was applied to the diffusion tensor image for the given subject, using FSL’s “vecreg”, in order to reorient each subject’s diffusion tensor image to standard space. These registered tensor images were then averaged (by scalar averaging tensor elements across subjects) to create a group-average diffusion tensor image, which was used to calculate the group FA image.

This atlas is available at Neurovault (https://neurovault.org/collections/LWTAAAST/).

### 2.5 Validation

The age-specific atlas was assessed by comparison with subject-specific tracts, delineated by applying the ROI-based method of delineating tracts, described above, to each validation subject. These tracts were transformed to the atlas space, by nonlinearly registering each subject’s FA image to the group FA template using FSL’s FNIRT (Andersson et al., 2007), and applying the resulting transformation to the segmented streamlines. We used three methods to assess accuracy of the atlas: i) volumetric overlap; ii) slice-wise correlation of FA measurements; and iii) correlation of whole-tract FA measurements. The same methods were also applied to the JHU atlas for comparison.

#### 2.5.1 Volumetric Overlap

To compare spatial similarity between normalised data we tested the volumetric overlap between the probabilistic atlas (age-specific or JHU) and each subject-specific tract by measuring the Dice score (Dice, 1945) over a range of probability thresholds. The amount of volumetric overlap between the atlas data and the subject-specific tract depends on both i) the quality of registration of the individual to the template, and ii) the agreement between the atlas data and the results from tractography in the individual. Thus, if the template is a closely matched target for registration, and if the underlying anatomy and diffusion process captured by the atlas is a good match to the validation subjects, we expect the Dice scores to be high.

#### 2.5.2 Slice-wise Correlation

We assessed the ability of the atlas to reproduce FA measurements from subject-specific tractography by calculating the mean FA in the tract in every slice along the major axis of each tract (coronal slices for tracts which project anterior/posterior; axial slices for tracts which project dorsal/ventral). In subject-specific tracts, average FA was calculated by taking the mean FA in all masked voxels. In the probabilistic atlases (age-specific or JHU), the FA was weighted by the probability in each voxel using the following equation:

(1) $$\text{FA}=\frac{\sum_{i}{\text{FA}}_{i}\times P_{i}}{\sum_{i}P_{i}}$$

where FA_i_ is the FA in voxel i and P_i_ is the probability in voxel i. We then calculated the correlation between the probabilistic FA and individual FA (see Section 2.7).

#### 2.5.3 Whole-tract Correlation

Whole-tract average FA was calculated in each subject, using both probabilistic and subject-specific tracts. Average FA was calculated in probabilistic tracts using equation (1) and in subject-specific tracts by averaging FA in all masked voxels. We then calculated the correlation between the probabilistic FA and individual FA (see Section 2.7).

#### 2.5.4 Healthy Brain Network (HBN) Data

In order to alleviate bias associated with using same-site scans for validation, we used an additional validation dataset obtained from the Healthy Brain Network (HBN, http://fcon_1000.projects.nitrc.org/indi/cmi_healthy_brain_network/) (Alexander et al., 2017), a data-sharing biobank from the Child Mind Institute. Scans were obtained from 15 subjects, aged 6-8 years, from release 7.0 from the CitiGroup Cornell Brain Imaging Centre dataset. These multi-shell DWI data were acquired on a Siemens 3 tesla Prisma scanner using using an echo-planar pulse sequence with the following parameters: TE = 100.2 ms; TR = 3320 ms; 81 slices; 1.8 x 1.8 x 1.8 mm resolution; multi-band acceleration factor = 3; b = 1,000 s mm^-2^ and b = 2,000 s mm^-2^, each with 64 directions, and one b = 0 image. Preprocessing and quality control pipelines were applied as described above, followed by calculation of FODs using multi-shell multi-tissue constrained spherical deconvolution (Jeurissen et al., 2014) and tractography as described above (see Section 2.4). This allowed validation using subjects scanned in a different scanner, and with different scanning parameters. To further alleviate bias associated with using the same tractography algorithm for atlas construction and validation we also ran tractography in this cohort using a deterministic tensor-based algorithm (Basser et al., 2000), in addition to the FOD-based tractography algorithm.

In order to assess whether the modern scanning protocols and preprocessing tools (such as the multi-shell acquisition and susceptibility-induced distortion correction with TOPUP) favoured the age-specific atlas over the JHU adult atlas (which was produced before these tools were available) we also assessed performance using a minimally processed version of the HBN validation cohort. To produce this dataset, we used single-shell data from each subject (using only the volumes acquired with b = 0 and b = 1,000 s mm^-2^). For each subject, FSL’s eddy_correct was used to register all volumes to the b = 0 image (but not correct for eddy current induced distortions). Tractography was then performed using the tensor-based algorithm described above.

To give an overall indication of the accuracy of the atlas in these datasets, we applied the whole-tract correlation method described above (see Section 2.5.3). For completeness, in-depth results of the volumetric overlap and slice-wise correlation for the HBN data are given in the Supplementary Materials.

### 2.6 CoolMRI Study

As a demonstration, the age-specific atlas was used to investigate tract-level differences in white matter FA between the case and control children of the CoolMRI study. In each of the tracts delineated by the age-specific atlas, the average whole-tract FA was calculated for each individual using equation (1). We then tested for group differences in whole-tract FA. Bilateral tracts were tested separately. For comparison, we repeated the analysis using the JHU adult atlas. In the absence of ground truth, only a qualitative comparison of results obtained with the two atlases was performed.

### 2.7 Statistical Analysis

To assess whether the age-specific atlas gave better volumetric agreement with subject-specific tracts than the JHU adult atlas, we performed a two-tailed, paired t-test comparing the peak Dice scores.

In the slice-wise FA analysis and whole-tract FA analysis, we measured the correlation between atlas measurements and individual measurements using a repeated measures correlation coefficient (Bland and Altman, 1995), which uses an analysis of variance to calculate the correlation coefficient between residuals of the repeated measurements. This method was used in slice-wise FA analysis to calculate the correlation coefficient without variation due to different subjects, and in the whole-tract FA analysis to calculate the correlation coefficient without variation due to different tracts.

For each validation method, we compared the correlation coefficient given by the age-specific atlas with that given by the JHU adult atlas, by applying Fisher’s z-transform to each correlation coefficient and estimating the 95% confidence intervals of the difference between these z-transformed correlation coefficients. The confidence intervals were estimated with a percentile bootstrap method (Wilcox and Muska, 2002). In the slice-wise correlations, a moving block bootstrap method was used to account for the spatial dependence of repeated measurements in each subject (Politis and Romano, 1992).

In the CoolMRI demonstration, Mann-Whitney U tests were applied to test for differences in the median FA between cases and controls in each tract, with Bonferroni correction applied to correct for family-wise error. Significant results have corrected p < 0.05.

## 3 Results

### 3.1 Participant Demographics

The CoolMRI study recruited 51 children, without CP, treated with TH for NE at birth and 43 control children matched for age, sex and SES (Lee-Kelland et al., 2020). Of the recruited children, 7 cases and 4 controls did not want to undergo scanning. A further 4 cases had incomplete data due to movement during the scan. Quality control led to the rejection of a further 6 cases and 2 controls. One further case and one control were rejected due to incorrect image volume placement. This left 33 cases and 36 controls. These controls were split into 28 for atlas construction and 8 for validation. Data for each set of participants, as well as for the 15 subjects from the HBN dataset, is shown in Table 1.

### 3.2 Atlas

**Figure 4:**
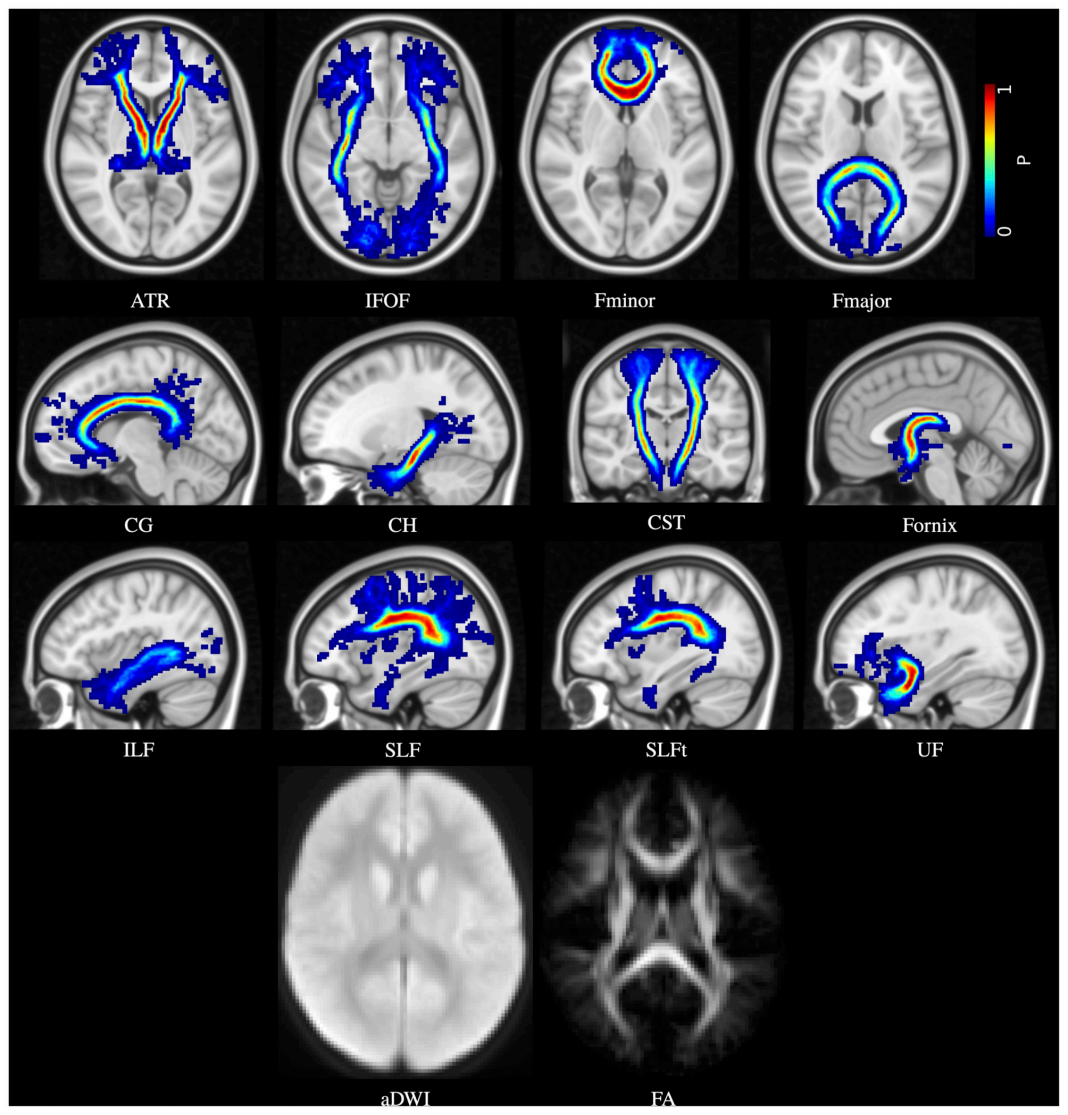
Age-specific probabilistic atlas for the 12 major white matter tracts: anterior thalamic radiation (ATR); inferior fronto-occipital fasciculus (IFOF); forceps minor (Fminor); forceps major (Fmajor); cingulate gyrus part of the cingulum (CG); hippocampal part of the cingulum (CH); cortico-spinal tract (CST); fornix; inferior longitudinal fasciculus (ILF); superior longitudinal fasciculus (SLF); temporal part of the superior longitudinal fasciculus (SLFt); and uncinate fasciculus (UF). Probabilities are indicated by the colour bar. Also shown are the average DWI (aDWI) and FA maps.

Figure 4 shows the probabilistic map for each tract, as well as the average DWI and FA images for the group of 28 children.

### 3.3 Validation

#### 3.3.1 Volumetric Overlap

**Figure 5:**
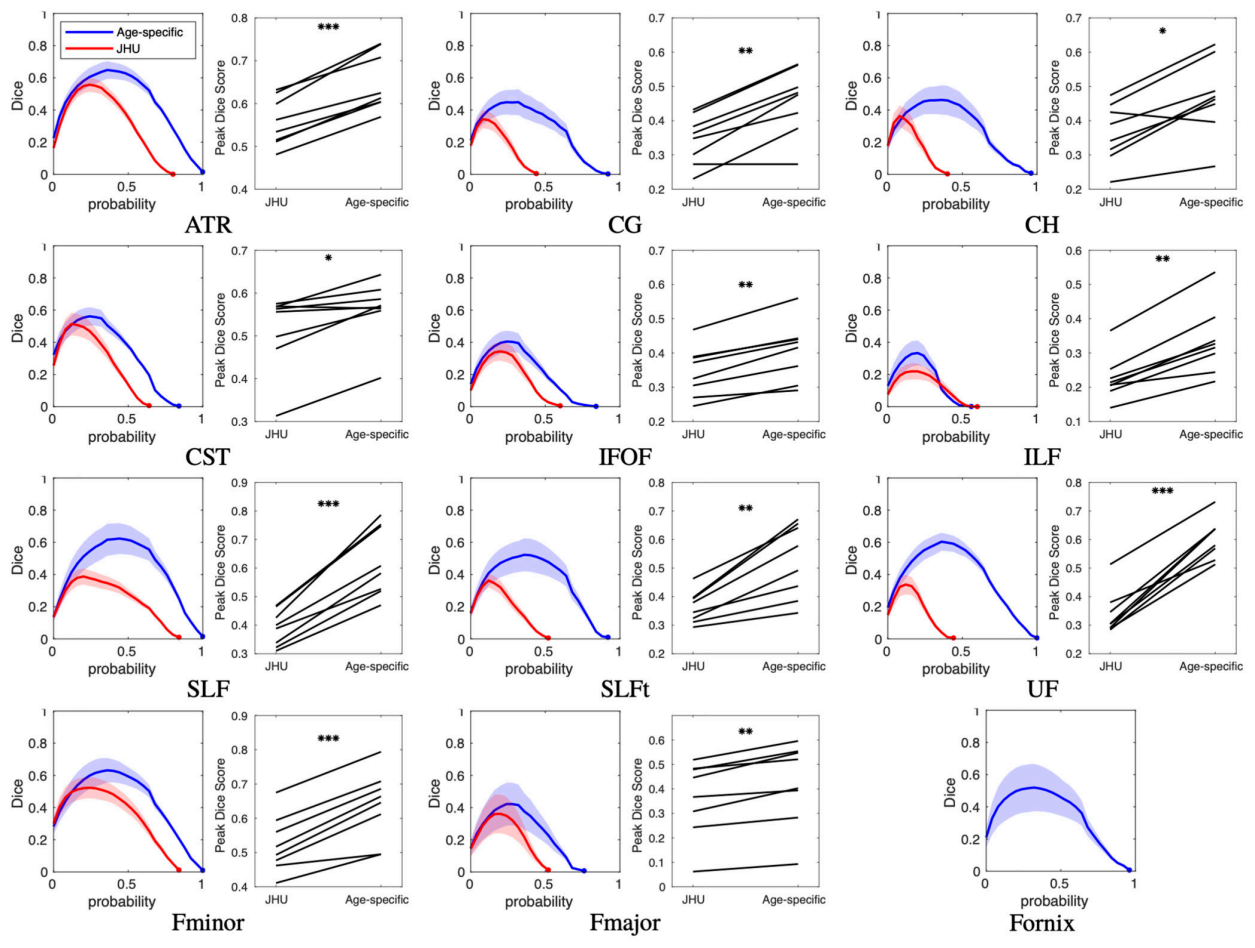
Same-site validation of tract overlap with “gold-standard” subject specific tract tracing. For each tract, the plot on the left shows the Dice score of volumetric overlap (y axis) against probability threshold (x axis) when using the age-specific atlas (blue) or the JHU adult atlas (red), with lines showing the mean score for the 8 validation subjects not included in the formation of the atlas, and shaded regions show the 95% confidence interval of the mean. Also shown for each tract is a paired plot of the peak Dice scores calculated with each atlas. P-values, given in Table S1, are indicated by: *p < 0.05; **p < 0.001; ***p < 0.0001. Note that the age-specific atlas outperformed the JHU (adult) atlas in all tracts. The tract representing the fornix is not available in the JHU atlas so only the new mask was tested.

The Dice score at a range of thresholds is plotted for each tract for the same-site validation data in Figure 5. The peak Dice scores for the age-specific atlas was significantly higher than for the JHU atlas in every tract (p < 0.05; see Table S1 for all p-values). The Dice scores for the HBN data are shown in Figures S1, S2 and S3.

#### 3.3.2 Slice-wise Correlation

**Figure 6:**
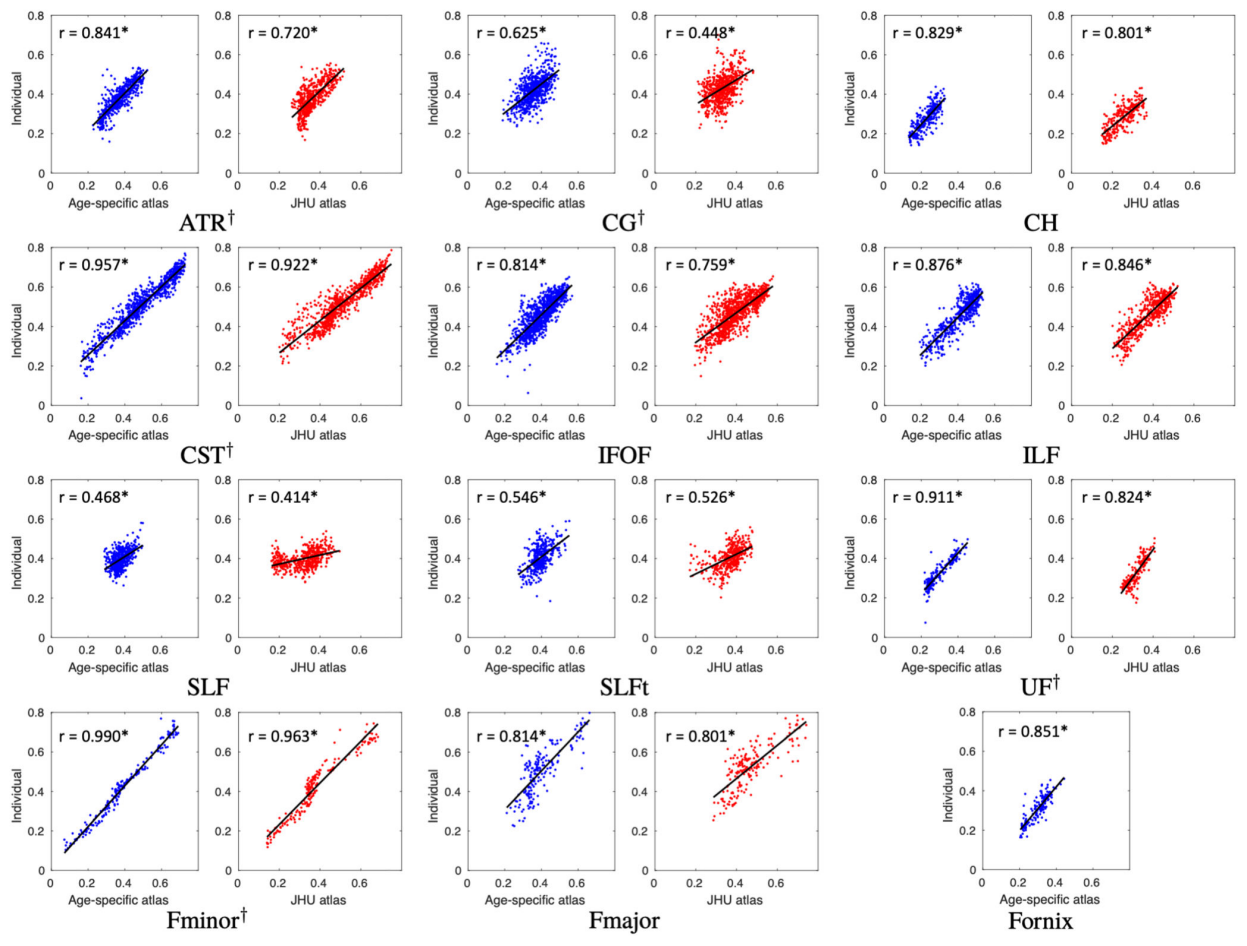
Same-site validation of slice FA values. Plots show slice FA measured from individually traced tracts (i.e. the “gold-standard”) plotted against corresponding values extracted from the age-specific and JHU atlases. Each plot shows a point for every slice in each of the 8 validation subjects and the regression. Correlation coefficients are shown on each plot, measured using a repeated measures correlation (Bland and Altman, 1995). All tracts exhibit higher correlation when measured with the age-specific atlas than with the JHU adult atlas. This difference is significant in the ATR, CG, CST, UF and Fminor, as indicated by † next to the tract abbreviation. Confidence intervals and regression parameters are given in Table S2. *p < 10^-20^.

The correlation between slice-wise FA measured by the age-specific atlas and that measured from subject-specific tracts is shown for the same-site validation data in Figure 6, with correlation coefficients measured using a repeated measures correlation (Bland and Altman, 1995). The correlations for the HBN data are shown in Figures S4, S5 and S6. A correlation coefficient of one indicates perfect slice-wise agreement between the gold-standard (FA extracted from subject-specific tracts) and the FA estimated for each tract by registration to the either age-specific or JHU adult atlas. In the same-site data, most tracts showed strong correlation between FA measured by the age-specific atlas and that measured from subject-specific tracts, with all tracts having r > 0.8 apart from the CG (r = 0.625), SLF (r = 0.468) and SLFt (r = 0.546). The correlation coefficient for the age-specific atlas was higher than for the JHU adult atlas in all tracts, and this difference was significant in the ATR, CG, CST, Fminor and UF (indicated by the 95% confidence intervals of the difference between z-transformed correlation coefficients, see Table S2).

#### 3.3.3 Whole-tract Correlation

**Figure 7:**
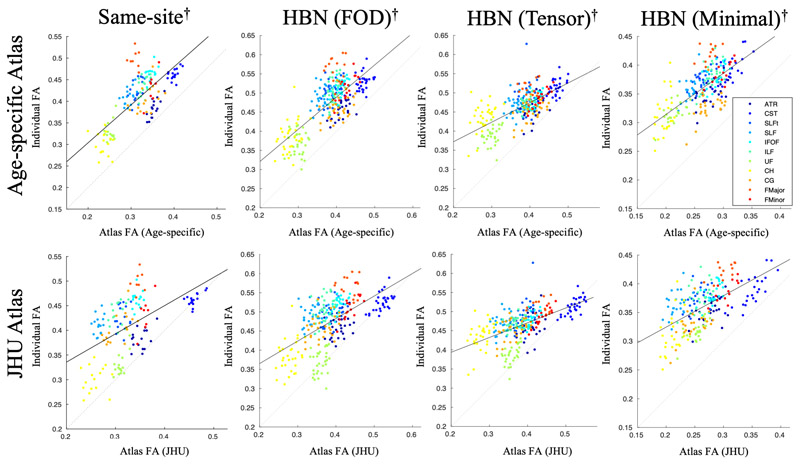
Comparison of mean FA extracted from whole tracts traced in individuals (“gold-standard”) and that estimated using each atlas. Whole-tract FA was measured by subject-specific tracing in the same-site validation data (left), the HBN data with FOD-based tractography (middle), and the HBN data with tensor-based tractography (right), then plotted against whole-tract FA measurements given by the age-specific atlas (top) or JHU adult atlas (bottom). The solid line shows the regression to all data points, and the dotted line represents exact equality between individual and the age-specific or JHU data. Repeated measures correlation coefficients are given in Table 2.

Figure 7 shows the whole-tract FA measured by the atlas plotted against that measured from subject-specific tracts for the same-site data, the HBN data with FOD-based tractography, the HBN data with tensor-based tractography, and the HBN data with minimal processing and tensor-based tractography. The fornix is not included in these plots to allow valid comparison with the JHU atlas. Correlation coefficients, and confidence intervals of the difference between z-transformed correlation coefficients, are shown in Table 2. The age-specific atlas gave significantly stronger correlation of whole-tract FA measurements than the JHU adult atlas in all validation datasets.

### 3.4 CoolMRI Study

**Figure 8:**
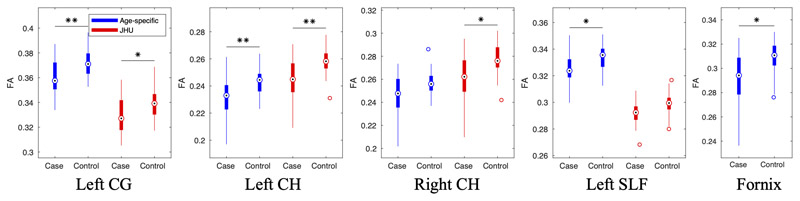
Box plots of significant differences in whole-tract average FA between children treated with TH for NE and healthy controls. Measurements obtained with both the age-specific atlas (blue) and the JHU adult atlas (red) are shown for tracts in which at least one of the atlases revealed significant differences between cases and controls; *p < 0.05, **p < 0.01, Bonferroni corrected. The fornix is not available in the JHU atlas so was only tested with the age-specific atlas. In each box, the point shows the median, the box shows the 25th to 75th percentiles, and the lines extend to the maximum and minimum data points, excluding outliers which are displayed as circles.

Numerous tracts in children treated with TH for NE had reduced FA compared to controls (see Table S6). After Bonferroni correction, only the left CG (p = 0.0056), left CH (p = 0.0081), left SLF (p = 0.0383), and fornix (p = 0.0121) had significantly reduced FA in cases compared to controls. The same analysis was run with the JHU atlas for comparison (see Table S7). Figure 8 shows box plots for both atlases for tracts in which at least one of the atlases revealed group differences in FA. Significant differences were found in the left SLF with the age-specific atlas but not the JHU adults atlas. Differences were found in the left CG and left CH with both atlases. Differences in the right CH were found with the JHU adult atlas but not with the age-specific atlas. Differences were found in the fornix with the age-specific atlas, but it is not available in the JHU atlas so could not be tested.

## 4 Discussion

This study introduces an age-specific probabilistic white matter atlas constructed from children aged 6-8 years, providing a method of delineating white matter tracts without tractography. We have shown that this atlas accurately delineates tracts in children from a same-site cohort, and a cohort from a different site, imaged with different scanner and acquisition protocol. The strong correlation between FA sampled by the atlas and that measured in each individual (i.e. the “gold standard”), at both a whole-tract level and slice-wise level, shows that the atlas provides an accurate estimate for the underlying white matter diffusion properties. Additionally, the Dice scores between tracts in the atlas and those delineated by subject-specific tracts were higher with the age-specific atlas than with the JHU adult atlas, demonstrating improved anatomical accuracy of the age-specific atlas. In these validation methods, the age-specific atlas performed better than simply registering to an existing adult white matter tract atlas, as is routinely done in the literature. As a demonstration, we applied the age-specific atlas to the CoolMRI study, revealing significantly reduced FA in several major white matter tracts in children treated with TH for NE at birth compared to healthy controls.

The correlation of whole-tract FA measured by the atlas with that measured in subject-specific tracts offers quantification of the performance of the atlas as a whole. In the same-site validation data, the HBN data with FOD-based tractography, the HBN data with tensor-based tractography and the HBN data with minimal processing and tensor-based tractography, the age-specific atlas exhibited stronger correlation with the individual measurements than for the JHU atlas (Figure 7, Table 2). This shows that the age-specific atlas can accurately characterise the distribution of tract-level FA in a study group, facilitating more sensitive group comparison and investigation of associations between these metrics and neuropsychological and behavioural measures.

Those tracts which exhibit low correlation between atlas and individual slice-wise FA measurements (namely the CG, SLF and SLFt) have very little spread in FA values, resulting in tightly grouped measurements with a low correlation coefficient (Figure 6). For these tracts, the Dice scores in Figure 5, as well as the tract-wise validation in Figure 7 demonstrate improved performance of the age-specific atlas at the level of whole tracts.

Long, thin tracts, such as the CST, IFOF and ILF, are particularly susceptible to partial volume effects when measuring volumetric overlap; a small radial translation will result in a large change to the Dice score. In these tracts, the high correlation in sampled FA values shows that the age-specific atlas gives accurate measurement of the tract diffusion properties.

Multi-site validation alleviates bias associated with using the same scanner for validation data and atlas construction, thus validation with the HBN data demonstrates that the age-specific atlas generalises to data from a different site, acquired with a different scanning protocol. In this dataset, the age-specific atlas gave better correlation of whole-tract FA measurements (Figure 7, Table 2). Additionally, the volumetric overlap in this dataset is significantly higher with the age-specific atlas than with the JHU adult atlas in all tracts apart from the CST and Fmajor, in which neither atlas performed significantly better than the other (Figure S1, Table S1). The age-specific atlas gave higher slice-wise correlations than the JHU adult atlas in all tracts; this difference was significant in the ATR, CST, Fminor, IFOF, ILF and UF (Figure S4, Table S3). There were no tests in which the JHU adult atlas performed significantly better in this dataset.

Further bias may be introduced by the use of the same tractography algorithm for both atlas generation and in estimating diffusion metrics for the validation data. Therefore, we also included a validation dataset in which subject-specific fibre bundles were delineated using a tensor-based tractography algorithm. Whereas the FOD-based tractography algorithm used to construct the age-specific atlas uses spherical deconvolution to find the peak FOD in the closest orientation to the propagating streamline, the tensor-based algorithm propagates the streamline along the principal eigenvector of the diffusion tensor at each step. This is comparable to the tensor-based tractography algorithm used in the construction of the JHU adult atlas, thus providing a conservative test case for validation. Despite this bias towards the JHU atlas, the age-specific atlas still gave stronger correlation of whole-tract FA measurements. In the tests of volumetric overlap (Figure S2, Table S1) and slice-wise correlation (Figure S5, Table S4) in this dataset, the age-specific atlas performed significantly better than the JHU adult atlas in at least one of these tests in six tracts (ATR, CH, ILF, UF, Fmajor, Fminor). In four tracts (CG, IFOF, SLF, SLFt) neither atlas performed significantly better in either test. In one tract (CST) the JHU atlas gave better volumetric overlap.

This introduces the question of how to provide the “gold-standard” of fibre tracking; the tensor-based algorithm was used in one of the HBN datasets in order to eliminate bias towards the age-specific atlas (by introducing bias towards the JHU adult atlas). However, this category of fibre tracking algorithm is widely acknowledged to give poor characterisation of diffusion in brain white matter due to its inability to resolve crossing fibres (Tournier et al., 2012; Behrens et al., 2007). Thus, the FOD-based algorithm used in the other validation datasets and in the construction of the atlas, which facilitates more comprehensive tracking due to its superior performance in regions of crossing fibres (Tournier et al., 2008), arguably gives a more accurate representation of the ground truth (i.e. the underlying white matter fibres). Therefore, when comparing the atlas to subject-specific fibre bundles in the validation data, the FOD-based algorithm likely gives a better indication of performance overall. Consequently, we believe the HBN data with tensor-based tractography provides a worst-case performance estimate, yet the age-specific atlas still out-performs the adult JHU atlas in many tests.

In future, as well as providing coverage of other age ranges, atlases could offer more extensive labelling of additional tracts and regions of white matter throughout development. A comprehensive database of fibre bundles across a range of ages, potentially constructed by applying automated tractography-based white matter tract segmentation protocols (Zhang et al., 2018; Wassermann et al., 2010; Verhoeven et al., 2009; Lawes et al., 2008) to data from population studies such as the Human Connectome Project (Van Essen et al., 2013), Developing Human Connectome Project (Hughes et al., 2017), or Baby Connectome Project (Howell et al., 2019), would allow study-specific atlases to be built from subjects matched to a given study cohort.

Applying the age-specific atlas to the CoolMRI study to investigate group differences in tract-level FA revealed selective reduction in FA, that was significantly reduced in the left CG, left CH, left SLF and the fornix (Table S6). For comparison, we performed the same analysis with the JHU adult atlas (Table S7). Figure 8 demonstrates the differences in FA measurements from the different atlases. These differences result in some tracts exhibiting group differences in one atlas but not the other (right CH and left SLF). Due to the absence of ground truth, these results do not support the use of one atlas over another. However, these results demonstrate that the two atlases can give differing outcomes in a case-control study. Quantitative results from the validation methods above indicate that the age-specific atlas gives more accurate delineation of white matter tracts in this age group than the JHU adult atlas, suggesting the CoolMRI results obtained with the age-specific atlas are more reliable.

Previous studies of neonates treated with TH for NE have investigated the relationship between white matter diffusion properties, measured in the first weeks following birth, and neurodevelopmental outcome at 2 years of age. These studies found a significant reduction in FA in infants with adverse outcomes, compared to those with favourable outcomes, in widespread areas of white matter including, but not limited to the corpus callosum, anterior and posterior limbs of the internal capsule, external capsule, fornix, cingulum, and ILF (Lally et al., 2019; Tusor et al., 2012). Many of these regions were also shown to have reduced FA in the CoolMRI cases, indicating that the early structural alterations resulting from the brain injury cause lasting changes to white matter development. These results also provide evidence for an underlying white matter deficit which manifests as neuropsychological differences seen at school-age (Lee-Kelland et al., 2020; Jary et al., 2019; Tonks et al., 2019). Further investigation is required to link these structural impairments to specific components of the cognitive and motor assessments, and to develop therapeutic intervention strategies.

## 5 Conclusions

The age-specific atlas provided by this study has been shown to robustly delineate white matter tracts in children aged 6-8 years. FA measurements sampled by the atlas correlate strongly with those measured by individual fibre tracking, allowing reliable investigation of white matter diffusion properties in cohorts. The closer agreement between FA measured in individually identified tracts and that estimated when registering to an age-specific atlas, suggests that such an approach would increase sensitivity to group differences, and is recommended for all studies performed in children.

## Supplementary Material

Supplementary Materials

## Figures and Tables

**Table 1:** Demographics of participants in the atlas dataset, same-site validation dataset, HBN validation dataset, and the CoolMRI dataset. Mean (range) is shown for age; median (range) is shown for SES and FSIQ in the CoolMRI cohort. Also shown are p-values of t-tests between atlas data and validation data for validation cohorts, and between cases and controls for the CoolMRI cohort. SES is defined as follows: A= upper middle class, B = middle class, C1 = lower middle class, C2 = skilled working class, D = working class, E = casual worker or unemployed. M/F = male/female; SES = socioeconomic status; FSIQ = full-scale intelligence quotient; HBN = Healthy Brain Network.

	Atlas	Same-site Validation		HBN Validation		CoolMRI		
			p		p	Cases	Controls	p
n =	28	8		15		33	36	
Age	7.0 (6.1-7.9)	7.0 (6.1-7.8)	0.9392	7.0 (6.0-7.9)	0.8684	6.9 (6.0-7.9)	7.0 (6.1-7.9)	0.5595
M/F	15/13	4/4	0.8776	9/6	0.7002	18/15	19/17	0.8894
SES						C1 (A-E)	B (A-D)	0.1568
FSIQ						93 (62-115)	108 (75-137)	<0.0001

**Table 2:** Validation of whole-tract FA correlations, corresponding to Figure 7. Columns show the parameters of the best-fit line y = mx + c and the correlation coefficient, r, between tract FA values from subject-specific fibre bundles and those from each atlas, measured using a repeated measures correlation (Bland and Altman, 1995). Also shown is the difference between the z-transform of the correlation coefficients for the age-specific atlas and the JHU atlas, and the 95% confidence intervals (CI) for this difference. Positive differences indicate a higher correlation with the age-specific atlas. These are shown for the same-site validation data, the HBN data with FOD-based tractography, the HBN data with tensor-based tractography, and the HBN data with minimal processing and tensor-based tractography. *p < 10^-10^. HBN = Healthy Brain Network; FOD = fibre orientation distribution; JHU = Johns Hopkins University.

	Age-specific Atlas			JHU Atlas			Difference between z-transformed correlation coefficients (95% CI)
Dataset	m	c	r	m	c	r	
Same-site	0.88	0.13	0.715*	0.57	0.22	0.536*	+=0.298 (+0.115, +0.300)
HBN (FOD)	0.84	0.15	0.781*	0.59	0.25	0.617*	+0.328 (+0.231, +0.412)
HBN (Tensor)	0.51	0.27	0.697*	0.39	0.32	0.595*	+0.176 (+0.087, +0.281)
HBN (Minimal)	0.72	0.17	0.661*	0.55	0.22	0.563*	+0.158 (+0.031, +0.268)

## Data Availability

This atlas is available at Neurovault (https://neurovault.org/collections/LWTAAAST/). The raw data that support the findings of this study are available upon reasonable request to the corresponding author.

## Impact Statement

We have developed a probabilistic white matter atlas for children aged 6-8 years. This atlas provides greater accuracy than using an adult white matter atlas, as assessed by independent validation studies. The atlas allows better delineation of white matter tracts without the need for computationally intensive, and potentially subjective, tractography methods; and will be of significant utility in the study of childhood white matter connectivity. This is particularly beneficial in a clinical setting, where the goal is to compare cohorts of children, as demonstrated by estimating white matter changes in children treated for hypoxic ischaemic injury at birth.
